# Psychometric evaluation of the PROMIS SD-SF-8b instrument in individuals experiencing vasomotor symptoms due to menopause

**DOI:** 10.1186/s12955-023-02206-x

**Published:** 2023-11-21

**Authors:** Neil M. Schultz, Antonia Morga, Emad Siddiqui, Stephanie E. Rhoten

**Affiliations:** 1 Medical Affairs, Astellas Pharma, 1 Astellas Way, Northbrook, IL 60062 USA; 2 Medical Affairs, Astellas Pharma Europe Ltd, Addlestone, UK; 3 Patient Centered Solutions, IQVIA, San Francisco, CA USA

**Keywords:** PROMIS, Psychometric, Sleep, Menopause, Vasomotor symptoms

## Abstract

**Background:**

Women with vasomotor symptoms (VMS) due to menopause frequently experience poor sleep quality. The Patient-Reported Outcomes Measurement Information System Sleep Disturbance – Short Form 8b (PROMIS SD-SF-8b) has been developed to assess sleep disturbance. The study objective was to use data from the fezolinetant SKYLIGHT 1 and 2 studies in individuals with VMS to assess the psychometric properties of the PROMIS SD-SF-8b.

**Methods:**

Individuals (aged ≥ 40–≤65 years) with moderate-to-severe VMS (≥ 7 hot flashes/day) were enrolled. Besides PROMIS SD-SF-8b, eight other patient-reported outcome (PRO) measures were used for the psychometric evaluation. All the PRO assessments were completed at weeks 4 and 12 during the treatment period and most were completed at baseline. Psychometric analyses included factor analysis and reliability, construct validity, and sensitivity to change assessments. The within-patient threshold for a clinically meaningful change in sleep disturbance was derived.

**Results:**

Overall, 1022 individuals were included from the SKYLIGHT 1 and 2 studies. Mean PROMIS SD-SF-8b total score at baseline was 26.80, which decreased to 22.68 at week 12, reflecting improved sleep disturbance. The confirmatory factor analysis supported the proposed PROMIS SD-SF-8b domain structure. Internal consistency was excellent, with Cronbach’s alpha values of 0.915 and 0.935 and a McDonald’s omega of 0.917. Item-to-item and item-total correlations were sufficient and moderate test-retest reliability was noted. The construct validity assessments showed that moderate Spearman rank correlations (*r*: 0.608 to 0.651) were observed between PROMIS SD-SF-8b total scores and measures of sleep disturbance and sleep-related impairment, and that significant differences were noted in the total scores across PRO categories. The responsiveness of PROMIS SD-SF-8b total scores was supported by the results from the correlations in change scores and comparisons of mean change scores by PRO categories. Statistically significant differences in mean scores were observed between responder and non-responder PRO groups. A PROMIS SD-SF-8b total score of 8 points was identified as the within-patient threshold to use to confirm a meaningful change in sleep disturbance.

**Conclusions:**

The psychometric properties of the PROMIS SD-SF-8b support its use to measure sleep disturbance in women with VMS due to menopause.

**Trial registration:**

ClinicalTrials.gov numbers: NCT04003155 and NCT04003142.

**Supplementary Information:**

The online version contains supplementary material available at 10.1186/s12955-023-02206-x.

## Background

Vasomotor symptoms (VMS), characterized by hot flashes and/or night sweats, are prevalent and bothersome for women experiencing menopause [1, 2]. Poor sleep quality, linked to VMS [3], is a major challenge during this period. Indeed, hot flashes affect sleep in 82% of women who have experienced menopause [4]. A screening survey also found that two-thirds of women who had experienced menopause had difficulties sleeping [5]. Frequency and severity of hot flashes had a linear relationship with sleep parameters in a 12-week study [6].

Menopausal hormone therapy (HT) remains the most recognizable approved option for treating VMS [7]. Despite proven efficacy [8], safety and tolerability concerns [9, 10], particularly in women with certain comorbidities [7], may limit HT use. The selective neurokinin 3 receptor antagonist, fezolinetant, is a nonhormonal treatment option approved by the US Food & Drug Administration for the treatment of moderate-to-severe VMS due to menopause [11]. A phase 2 study demonstrated that fezolinetant significantly reduced the frequency and severity of moderate-to-severe VMS versus placebo [12]. In another study, fezolinetant improved sleep quality, using the Leeds Sleep Evaluation Questionnaire, versus placebo at 4, 8, and 12 weeks [13]. SKYLIGHT 1 and 2 were two phase 3 studies that investigated the efficacy and safety of fezolinetant and included sleep disturbance endpoints [14, 15].

The Patient-Reported Outcomes Measurement Information System (PROMIS) is a set of patient-centered instruments that evaluate physical, mental, and social health [16]. PROMIS can be used within the general population and those with chronic conditions. The PROMIS Sleep Disturbance – Short Form 8b (PROMIS SD-SF-8b) was developed from PROMIS as a sleep disturbance assessment. The measure evaluates: difficulties and concerns with falling asleep, staying asleep, and getting enough sleep; and perceptions on the quality and satisfaction of sleep. Previous investigations analyzed the psychometric properties of PROMIS SD-SF-8b in the general population [17] and its qualitative features in individuals experiencing moderate-to-severe VMS [18].

No prior studies have assessed the psychometric properties of PROMIS SD-SF-8b in individuals with moderate-to-severe VMS. These properties need to be evaluated to support its use in clinical trials. Using Food and Drug Administration guidance [19, 20], we utilized pooled data from the SKYLIGHT 1 and 2 studies to assess the psychometric properties of the PROMIS SD-SF-8b in individuals with moderate-to-severe VMS due to menopause.

## Methods

### Participants

The SKYLIGHT 1 and 2 study methodologies (NCT04003155 and NCT04003142, respectively) have been published previously [14, 15]. Briefly, SKYLIGHT 1 and 2 were identical, phase 3, randomized, placebo-controlled, double-blind studies conducted in Europe and North America that investigated fezolinetant efficacy and safety. Individuals who were female at birth (≥ 40–≤65 years) with moderate-to-severe VMS (seven hot flashes/day) were enrolled. The participants were randomized to receive once-daily doses of fezolinetant 30 mg, fezolinetant 45 mg, or placebo (1:1:1) during a 12-week double-blind period. Completers entered a 40-week active treatment extension, where fezolinetant-treated individuals continued their initial dose, while the placebo group was re-randomized to receive fezolinetant 30 mg or 45 mg.

### PROMIS SD-SF-8b

The PROMIS SD-SF-8b comprises eight items selected from the PROMIS bank to measure sleep disturbance over the past 7 days [21, 22]. Total score is calculated by summing the items (range: 8–40; higher score: more disturbed sleep). If some items were not completed, it was not possible to calculate the total score and the result was considered missing.

### Additional patient-reported outcome (PRO) measures

Eight PRO measures were used to evaluate PROMIS SD-SF-8b; VMS episodes captured using an electronic diary; PROMIS Sleep-Related Impairment – Short Form 8a (PROMIS SRI-SF-8a; eight items); Menopause-Specific Quality of Life (MENQOL) questionnaire (29 items); Patient Global Impression of Severity Sleep Disturbance (PGI-S SD) measure (single item); Patient Global Impression of Change Sleep Disturbance (PGI-C SD) measure (single item); Patient Global Impression of Change Vasomotor Symptoms (PGI-C VMS) measure (single item); EQ-5D-5 L (five questions) including EQ Visual Analog Scale (VAS); and Work Productivity and Activity Impairment questionnaire specific to Vasomotor Symptoms (WPAI-VMS; six items; Additional file 1: Further Methods) [23–26]. Given the nature of these specific measures, it is likely that useful associations can be derived following the psychometric evaluation of the PROMIS SD-SF-8b.

All the PRO assessments were self-administered during the site visit before any other study procedures were performed. The assessments were conducted electronically using a tablet. All assessments were completed at baseline and weeks 4 and 12, apart from the PGI-C assessments (weeks 4 and 12 only because PGI-C analyzes change from baseline). The assessments were consistently conducted in the following order: PGI-C VMS, PROMIS SD-SF-8b, PGI-S SD, PGI-C SD, PROMIS SRI-SF-8a, MENQOL, EQ-5D-5 L, and WPAI-VMS.

### Descriptive analyses

Completion rate was calculated by dividing the number of individuals who completed the PROMIS SD-SF-8b at each visit by the number of individuals in the full analysis set (FAS).

Descriptive statistics were provided for the PROMIS SD-SF-8b total score and the number of individuals who selected each answer. Baseline floor and ceiling effects were investigated, which were defined as > 20% of the responses for the lowest/least severe or highest/most severe options, respectively (calculated as 100% divided by the number of options [five]).

### Psychometric evaluation

At baseline, a confirmatory factor analysis (CFA) was performed for the PROMIS SD-SF-8b items. As the data were categorical, the factor structure was defined using the unweighted least squares method. This method does not assume multivariate normality and is appropriate for ordinal data with ≤5 categories, like the PROMIS SD-SF-8b. Goodness-of-fit measures were developed to evaluate the model; standardized root mean residual (SRMR) and non-normed fit index (NNFI). To demonstrate good fit, the SRMR had to be below the recommended threshold of 0.08 and the NNFI had to be above 0.95 [27].

Internal consistency was assessed using Cronbach’s alpha coefficient (values ≥ 0.70: acceptable reliability [28]). Alpha-if-item-deleted results were derived, and McDonald’s omega was calculated (values > 0.80: good internal consistency) [29, 30]. Item-to-item correlations were calculated at baseline and item-total correlations were calculated at baseline and week 12. Among items expected to measure the same construct, correlations should fall in the 0.4 to 0.8 range [31]. For item-total correlations, however, too large a coefficient (e.g., ≥ 0.80) might suggest redundancy (e.g., one item is a restatement of another). The correlation between individual items and the total score omitting the item is provided for the item-total correlations. Test-retest reliability was evaluated using a two-way mixed, absolute agreement, single measure intraclass correlation coefficient (ICC; values 0.50–0.90: moderate-to-good reliability, values > 0.90: excellent reliability [32]). Test was defined as baseline and retest was defined as week 4. Stable individuals were required and were defined as participants reporting no change in PGI-S SD over this time.

Construct validity was evaluated using Spearman coefficients for convergent validity and analysis of variance with orthogonal planned comparisons for known-groups validity. For convergent validity, correlations were examined between PROMIS SD-SF-8b scores and other PRO measures at baseline. At least moderate correlations between overall/scale scores of similar constructs (*r*: >0.40) were expected [31]. Known-groups validity was assessed by examining baseline PROMIS SD-SF-8b scores across PGI-S SD categories to test whether PROMIS scores differed between adjacent PGI-S groups (i.e., “no problems” versus “mild problems”). The known-groups was the independent variable and PROMIS SD-SF-8b scores were the dependent variables.

Sensitivity to change was examined using Spearman correlations and analysis of covariance (ANCOVA). Correlations between changes in PROMIS SD-SF-8b and several PRO scores from baseline to week 12 were calculated. Concurrent improvement in PRO measures would result in moderate-to-strong correlations. In separate ANCOVA models controlled for baseline values, changes in PROMIS SD-SF-8b scores from baseline to week 12 were assessed for individuals reporting improvement (responders) versus individuals reporting no change/worsening (non-responders) for the PGI-S SD and PGI-C SD. The groups were identified using the PGI-S SD change from baseline to week 12 results and the PGI-C SD response at week 12.

As recommended by the Food and Drug Administration [19, 20], thresholds for meaningful within-patient change for PROMIS SD-SF-8b were estimated using anchor-based approaches, supplemented with distribution-based estimates and receiver operating characteristic (ROC) curves. Meaningful within-patient change was evaluated using the PGI-S SD and PGI-C SD as anchors. Spearman correlations between changes in anchor (Additional file 1: Table S1) and PROMIS SD-SF-8b scores were assessed between baseline and weeks 4 and 12 (suitable anchor correlation: >0.30 [33]). The distribution-based estimates included the effect size (Cohen’s d), half the baseline standard deviation (SD), and standard error of measurement (SEM; SD*√[1–*r*], where *r* equals internal consistency). Interpretation was based on conventional benchmarks (small [0.2], medium [0.5], or large [0.8] effect size [34]). For the anchor-based approach, descriptive statistics for change between baseline and weeks 4 and 12 were calculated based on improvement, no change, or worsening on the anchors. Multiple estimates are presented for each score owing to the multiple anchors and methods used to estimate responder definitions. Using the PGI-S SD and PGI-C SD, the thresholds for meaningful within-patient change were defined as a 2-point improvement and feeling “moderately better”, respectively. Mean changes in PROMIS SD-SF-8b for the PGI-S SD and PGI-C SD anchors were also calculated for the other change categories (Additional file 1: Table S1). For the triangulation, the thresholds were selected based on the within-patient change for anchor improvement categories (PGI-S SD: 2-point improvement, PGI-C SD: “moderately better”), sufficient anchor correlations ≥ 0.30, and the lower 95% confidence interval (CI) estimates for the individuals experiencing “no change” on the anchors (the lower CI: greatest improvement). ROC curves were consulted as these provide the best estimate of the point that divides individuals who report minimal/little/no change and those who report change. Due to the variability in the change estimates between the PGI-S SD and PGI-C SD anchors, the selected thresholds were in the middle of the range. For the anchor-based approach (ROC curve), sensitivity and specificity were calculated to characterize the association between PROMIS SD-SF-8b changes and anchor improvement. ROC curves were derived using logistic regression analyses. For this analysis, the change groups in Additional file 1: Table S1 were collapsed into two groups: improvement and minimal/no improvement (Additional file 1: Table S2). Responder status was the dependent variable and change from baseline in PROMIS SD-SF-8b score was the independent variable. The clinically meaningful threshold was defined by the change value corresponding to the cutpoint in the ROC space that minimizes the sum of squares of (1-sensitivity) and (1-specificity), closest to the top-left corner (1,0) of the ROC space [35].

### Statistical analyses

All analyses were conducted using pooled treatment data from SKYLIGHT 1 and 2. All PRO analyses were performed on the FAS (all randomized individuals who received ≥ 1 dose of study drug). Statistical comparisons involved two-sided tests at the α = 0.05 level. For point estimates, 95% CIs were used. All data processing was performed using SAS Version 9.3 or higher (SAS Institute, Cary, North Carolina, USA).

## Results

### Demographics and completion rates

Overall, 1022 individuals were included in the FAS from SKYLIGHT 1 and 2. Average age was similar in both studies (mean [SD] – SKYLIGHT 1: 54.4 [4.9] years, SKYLIGHT 2: 54.3 [5.0] years) and most participants were white (SKYLIGHT 1: 82.7%, SKYLIGHT 2: 79.4%) [14, 15]. Time since onset of hot flashes was also similar in both studies (mean [range] – SKYLIGHT 1: 77.1 [1–422] months, SKYLIGHT 2: 80.0 [2–396] months) and the frequency of VMS at baseline was similar for all three treatment groups (pooled data for each group; mean [SD] – placebo: 11.0 [4.5] episodes, fezolinetant 30 mg: 10.9 [4.8] episodes, fezolinetant 45 mg: 11.1 [6.5] episodes). Furthermore, the majority of the participants were enrolled from North America (all randomized participants; SKYLIGHT 1: 351 [66.6%] participants, SKYLIGHT 2: 356 [71.1%] participants) in comparison with Europe (SKYLIGHT 1: 176 [33.4%] participants, SKYLIGHT 2: 145 [28.9%] participants).

In total, 1019 (99.7%) participants had baseline data, with high completion rates of 91.3% (933/1022) and 84.6% (865/1022) at weeks 4 and 12, respectively.

### PROMIS SD-SF-8b scores

**Fig. 1:**
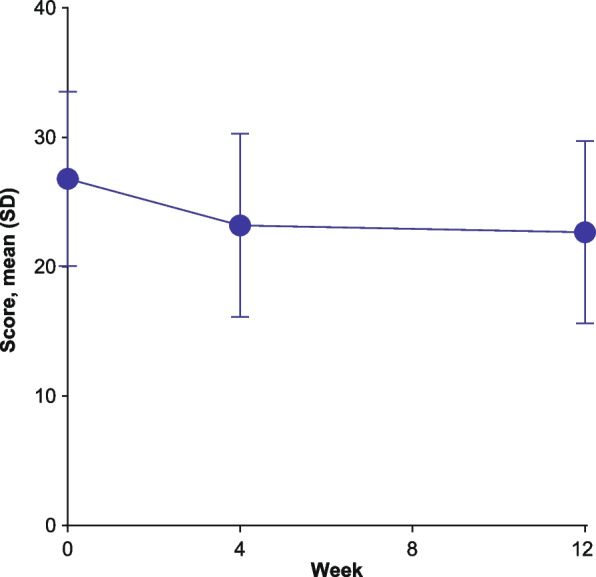
PROMIS SD-SF-8b total score over time. PROMIS SD-SF-8b, Patient-Reported Outcomes Measurement Information System Sleep Disturbance – Short Form 8b; SD, standard deviation. Higher scores indicate worse sleep disturbance. Participant numbers: *N* = 1019 (baseline), *N* = 933 (week 4), and *N* = 865 (week 12)

The mean PROMIS SD-SF-8b total score at baseline was 26.80, which improved to 23.21 and 22.68 at weeks 4 and 12, respectively (Fig. 1). The same findings were observed for each individual item, with proportionally more individuals reporting the lowest/least severe response (Additional file 1: Table S3). At baseline, slight ceiling effects were found for items 2 (25.3%) and 3 (26.7%) and floor effects were found for item 4 (22.1%).

### Psychometric evaluation

Using baseline data, the CFA supported the proposed PROMIS SD-SF-8b domain structure (Table 1). Good fit was demonstrated by an SRMR of 0.047 and a NNFI of 0.990, supporting the scoring for the total score. The item factor loadings with the general domain were consistently large (0.510 to 0.870).

**Table 1:** Confirmatory factor analysis

Hypothesized model structure	Statistic^a^	PROMIS SD-SF-8b total score (*N* = 1018)
All items constitute one factor	SRMR	0.047
	NNFI	0.990
**Factor loadings**		
Item 1: my sleep was restless		0.763
Item 2: I was satisfied with my sleep		0.812
Item 3: my sleep was refreshing		0.751
Item 4: had difficulty falling asleep		0.510
Item 5: had trouble staying asleep		0.774
Item 6: had trouble sleeping		0.823
Item 7: got enough sleep		0.757
Item 8: my sleep quality was…		0.870

*NNFI *Non-normed fit index, *PROMIS SD-SF-8b *Patient-Reported Outcomes Measurement Information System Sleep Disturbance – Short Form 8b, *SRMR S*tandardized root mean residual

^a^Rules for assessing the model fit – SRMR: ≤0.08, NNFI: ≥ 0.95 [27]

Internal consistency was excellent for the PROMIS SD-SF-8b (Table 2), with Cronbach’s alpha values of 0.915 and 0.935 at baseline and week 12, respectively, and a McDonald’s omega of 0.917. The alpha coefficients were also excellent when each item was individually deleted, with values between 0.895 and 0.941. Item-to-item correlations at baseline were sufficient without typically suggesting redundancy, with results between 0.414 and 0.778 (Additional file 1: Table S4). One weak correlation of 0.357 was observed between items 3 and 4 and two high correlations of 0.829 and 0.806 between items 2 and 3 and items 5 and 6, respectively. Item-total correlations were sufficient for the PROMIS SD-SF-8b score at baseline and week 12 (Table 3). Strong correlations between item and PROMIS SD-SF-8b scores omitting the item were frequently observed at both baseline (*r* range: 0.529–0.789) and week 12 (0.606–0.795). However, potential redundancy was also observed for some correlations at both baseline (*r* range: 0.812–0.865) and week 12 (0.819–0.891). Moderate test-retest reliability was found for PROMIS SD-SF-8b scores (ICC: 0.662; 95% CI: 0.598, 0.717). Values of 0.50–0.90 represent moderate-to-good reliability [32].

**Table 2:** Internal consistency reliability analysis

Item	Baseline		Week 12	
	Alpha item deleted	Cronbach’s alpha (95% CI)	Alpha item deleted	Cronbach’s alpha (95% CI)
PROMIS SD-SF-8b total score		0.915 (0.906, 0.922)		0.935 (0.928, 0.941)
Item 1: my sleep was restless	0.903		0.930	
Item 2: I was satisfied with my sleep	0.899		0.924	
Item 3: my sleep was refreshing	0.904		0.924	
Item 4: had difficulty falling asleep	0.922		0.941	
Item 5: had trouble staying asleep	0.902		0.925	
Item 6: had trouble sleeping	0.898		0.920	
Item 7: got enough sleep	0.903		0.926	
Item 8: my sleep quality was…	0.895		0.920	

Cronbach’s alpha is calculated between each item and the total score omitting the item

*CI* Confidence interval; *PROMIS SD-SF-8b* Patient-Reported Outcomes Measurement Information System Sleep Disturbance – Short Form 8b

**Table 3:** Item-total correlation analysis

Item	Baseline	Week 12
Item 1: my sleep was restless	0.748	0.753
Item 2: I was satisfied with my sleep	0.789	0.834
Item 3: my sleep was refreshing	0.731	0.834
Item 4: had difficulty falling asleep	0.529	0.606
Item 5: had trouble staying asleep	0.760	0.819
Item 6: had trouble sleeping	0.812	0.885
Item 7: got enough sleep	0.745	0.795
Item 8: my sleep quality was…	0.865	0.891

Polyserial correlation coefficients were calculated between each item and the total score omitting the item

The convergent validity results demonstrated moderate Spearman rank correlations between PROMIS SD-SF-8b scores and PGI-S SD (*r*: 0.651) and PROMIS SRI-SF-8a (*r*: 0.608) at baseline (Table 4). Low absolute correlations were observed with the frequency and severity of VMS (*r*: 0.114 and 0.158, respectively) and EQ VAS (*r*: − 0.254). Correlations were also low between PROMIS SD-SF-8b scores and the WPAI questionnaire, with similar results typically noted for the separate components (*r* range: 0.219–0.230). The only exception was WPAI absenteeism, which displayed a lower correlation (*r*: 0.073). Known-groups validity showed significant differences in PROMIS SD-SF-8b scores across PGI-S SD categories (*p* < 0.0001; Table 5). As expected, lower (better) PROMIS SD-SF-8b scores were observed for individuals with better PGI-S SD scores. The contrast category results showed that PROMIS SD-SF-8b scores were significantly different between adjacent PGI-S SD categories (*p* < 0.0001).

**Table 4:** Convergent validity: correlations between PROMIS SD-SF-8b total score and assessments of related constructs

Measure	PROMIS SD-SF-8b total score
PGI-S SD (*N* = 1019)	0.651
PROMIS SRI-SF-8a (*N* = 1019)	0.608
MENQOL (*N* = 1017)	0.380
WPAI activity impairment (*N* = 1016)	0.230
WPAI presenteeism (*N* = 621)	0.221
WPAI overall work productivity loss (*N* = 621)	0.219
Severity of VMS (*N* = 1019)	0.158
Frequency of VMS (*N* = 1019)	0.114
WPAI absenteeism (*N* = 622)	0.073
EQ VAS (*N* = 1016)	– 0.254

Spearman rank correlation coefficients are presented. For the PROMIS SRI-SF-8a instrument, only the total score is used for the correlation. For the MENQOL instrument, only the vasomotor subscale score is used for the correlation

*MENQOL *Menopause-Specific Quality of Life, *PGI-S SD *Patient Global Impression of Severity Sleep Disturbance, *PROMIS SD-SF-8b *Patient-Reported Outcomes Measurement Information System Sleep Disturbance – Short Form 8b, *PROMIS SRI-SF-8a *Patient-Reported Outcomes Measurement Information System Sleep-Related Impairment – Short Form 8a,  *VAS *Visual Analog Scale, *VMS *Vasomotor symptoms, *WPAI *Work Productivity and Activity Impairment

**Table 5:** Known-groups validity: PROMIS SD-SF-8b total score by PGI-S SD categories at baseline

PGI-S SD categories	*N*	LS mean (SE)	95% CI	*p* value	Contrast *p* value
No problems	70	16.94 (0.60)	15.76, 18.13	< 0.0001	
Mild problems	233	22.47 (0.33)	21.82, 23.12		< 0.0001
Moderate problems	484	27.31 (0.23)	26.86, 27.76		< 0.0001
Severe problems	232	33.04 (0.33)	32.39, 33.70		< 0.0001

*p* value is based on analysis of variance comparisons with alpha = 0.05 level. Contrast *p* value comparisons examined adjacent PGI-S SD groups, i.e., “no problems” versus “mild problems”, “mild problems” versus “moderate problems”, and “moderate problems” versus “severe problems”

*CI *Confidence interval, *LS L*east squares, *PGI-S SD *Patient Global Impression of Severity Sleep Disturbance, *PROMIS SD-SF-8b *Patient-Reported Outcomes Measurement Information System Sleep Disturbance – Short Form 8b, *SE S*tandard error

Results from correlations in change scores and comparisons of change scores by PGI-S SD and PGI-C SD categories supported the responsiveness of PROMIS SD-SF-8b. Moderate-to-strong correlations (*r*: >0.30) were observed between the change from baseline in PROMIS SD-SF-8b scores and PROMIS SRI-SF-8a (0.663), PGI-S SD (0.616), PGI-C SD (0.526), MENQOL vasomotor domain (0.458), and PGI-C VMS (0.373). Lower correlations were observed for the frequency of VMS (0.280) and EQ VAS (– 0.221). Statistically significant differences in score changes were observed between responder and non-responder groups according to change in PGI-S SD and PGI-C SD (*p* < 0.0001; Table 6). Responders (individuals reporting improvement at week 12) using the PGI-S SD or PGI-C SD categories reported greater reductions in PROMIS SD-SF-8b scores versus non-responders (individuals reporting no change/worsening).

**Table 6:** Sensitivity to change: analysis by PGI-S SD and PGI-C SD change groups

Anchor	Groups	*N*	LS mean (SE)	95% CI	*p* value
PGI-S SD	Responders	445	– 7.27 (0.27)	– 7.80, − 6.74	< 0.0001
	Non-responders	419	– 0.65 (0.28)	– 1.20, − 0.11	
PGI-C SD	Responders	590	– 6.33 (0.23)	– 6.78, − 5.89	< 0.0001
	Non-responders	272	0.91 (0.33)	0.25, 1.57	

*p* value: analysis of covariance with alpha = 0.05 level comparing “responders” and “non-responders”. PGI-S SD: “responders” = ≥ 1-point decrease from baseline to week 12, “non-responders” = all others. PGI-C SD: “responders” = any improvement at week 12, “non-responders” = all others

*CI *Confidence interval, *LS L*east squares, *PGI-C SD *Patient Global Impression of Change Sleep Disturbance, *PGI-S SD *Patient Global Impression of Severity Sleep Disturbance, *SE S*tandard error

The PGI-S SD and PGI-C SD were used as anchors for the PROMIS SD-SF-8b, with correlations between changes in PROMIS SD-SF-8b scores and the anchor scores varying between 0.526 and 0.616 (Additional file 1: Table S5). Some variability was observed in the distribution-based estimates for the PROMIS SD-SF-8b. The 0.5 SD at baseline was 3.37, SEM was 1.97, and a medium effect size of − 0.60 was observed. Mean changes for each group were generally consistent between weeks 4 and 12 within the anchors (Table 7). The estimates using the PGI-S SD anchor were typically larger (PROMIS SD-SF-8b score improvement: − 6.07 to − 19.00) than those using the PGI-C SD (– 3.59 to − 10.98). Individuals reporting “no change” for both anchors and timepoints had a score change of − 1.17 to 0.33 and those who reported worsening had a change of 2.82 to 10.56. The areas under the curve for the PROMIS SD-SF-8b scores were sufficiently above the recommended threshold (Additional file 1: Table S6). The results ranged between 0.76 and 0.84 and the thresholds for the total score were − 7 and − 8 using the PGI-S SD anchor and − 4 using the PGI-C SD. For the responder definition, the proposed thresholds were selected according to the range of within-patient change defined for anchor improvement categories (PGI-S SD: 2-point improvement, PGI-C SD: “moderately better”), sufficient anchor correlations (all ≥ 0.50), and the lower 95% CI estimates for the individuals experiencing “no change” on the anchors. The anchor-based estimates for the PROMIS SD-SF-8b score were − 11.28 and − 12.25 using the PGI-S SD (median: − 11.5 and − 12.0) and − 5.87 and − 5.93 using the PGI-C SD (median: − 6.0; Table 7). The largest thresholds from the ROC analyses were − 7 and − 8 points using the PGI-S SD and supported the lower-to-middle range of the anchor-based results. The triangulation of estimates suggested a range of − 6 to − 12 points and a threshold of − 8 points for the PROMIS SD-SF-8b. A PROMIS SD-SF-8b score of 8 points (range: 6 to 12 points) was therefore identified as the within-patient threshold to use to confirm a meaningful change in sleep disturbance. This result is higher than the distribution-based results (0.5 SD at baseline: 3.37, SEM: 1.97) and the largest estimate of individuals reporting no change on the anchors (– 1.78).

**Table 7:** Mean change in PROMIS SD-SF-8b total score: PGI-S SD and PGI-C SD categories

**Categories**	***N***	**Mean change**	**Median change**	**95% CI**
**PGI-S SD**				
**Baseline to week 4**				
All categories				
Improved 3 points	6	– 19.00	– 20.50	– 29.09, – 8.91
**Improved 2 points**	**102**	**– 12.25**	**– 12.00**	**– 13.66, – 10.85**
Improved 1 point	341	– 6.07	– 6.00	– 6.65, – 5.49
No change	377	– 1.02	– 1.00	– 1.58, – 0.46
Worsened 1 point	97	3.70	4.00	2.53, 4.88
Worsened 2 points	9	10.56	9.00	6.58, 14.53
Worsened 3 points	0			
Collapsed categories				
Improved ≥ 2 points (collapsed)	108	– 12.63	– 13.00	– 14.04, – 11.22
Worsened ≥ 2 points (collapsed)	9	10.56	9.00	6.58, 14.53
** Baseline to week 12**				
All categories				
Improved 3 points	12	– 18.33	– 22.00	– 23.43, – 13.23
** Improved 2 points**	**120**	**– 11.28**	**– 11.50**	**– 12.51, – 10.04**
Improved 1 point	313	– 6.30	– 6.00	– 6.92, – 5.68
No change	311	– 1.17	– 1.00	– 1.78, – 0.57
Worsened 1 point	98	3.35	3.00	2.16, 4.53
Worsened 2 points	10	7.30	7.00	2.73, 11.87
Worsened 3 points	0			
Collapsed categories				
Improved ≥ 2 points (collapsed)	132	– 11.92	– 12.00	– 13.16, – 10.67
Worsened ≥ 2 points (collapsed)	10	7.30	7.00	2.73, 11.87
**PGI-C SD**				
** Baseline to week 4**				
All categories				
Much better	160	– 10.98	– 10.00	– 12.12, – 9.84
** Moderately better**	**152**	**– 5.93**	**– 6.00**	**– 6.86, – 4.99**
A little better	265	– 3.80	– 4.00	– 4.51, – 3.09
No change	291	0.33	0.00	– 0.32, 0.98
A little worse	39	2.82	4.00	0.52, 5.12
Moderately worse	16	3.38	4.00	0.86, 5.89
Much worse	8	4.63	5.00	0.09, 9.16
Collapsed categories				
Much or moderately better	312	– 8.52	– 8.00	– 9.31, – 7.73
Moderately worse or much worse	24	3.79	4.00	1.73, 5.85
Improvement	577	– 6.35	– 6.00	– 6.92, – 5.78
No change	291	0.33	0.00	– 0.32, 0.98
Worsening	63	3.19	4.00	1.60, 4.78
** Baseline to week 12**				
All categories				
Much better	189	– 9.69	– 10.00	– 10.71, – 8.67
**Moderately better**	**170**	**– 5.87**	**– 6.00**	**– 6.77, – 4.97**
A little better	231	– 3.59	– 3.00	– 4.45, – 2.74
No change	198	– 0.56	0.00	– 1.38, 0.26
A little worse	37	3.00	2.00	1.20, 4.80
Moderately worse	29	4.03	4.00	1.72, 6.35
Much worse	8	6.63	4.50	1.90, 11.35
Collapsed categories				
Much or moderately better	359	– 7.88	– 8.00	– 8.59, – 7.17
Moderately worse or much worse	37	4.59	4.00	2.59, 6.60
Improvement	590	– 6.20	– 6.00	– 6.77, – 5.63
No change	198	– 0.56	0.00	– 1.38, 0.26
Worsening	74	3.80	3.00	2.47, 5.13

Bolded categories were used for the triangulation of meaningful change thresholds

* CI* Confidence interval, *PGI-C SD* Patient Global Impression of Change Sleep Disturbance, *PGI-S SD* Patient Global Impression of Severity Sleep Disturbance, *PROMIS SD-SF-8b* Patient-Reported Outcomes Measurement Information System Sleep Disturbance – Short Form 8b

## Discussion

This study evaluated the psychometric properties, sensitivity to change, and clinically meaningful within-patient change of the PROMIS SD-SF-8b instrument in individuals with moderate-to-severe VMS. Acceptable psychometric properties were noted for the PROMIS SD-SF-8b, and a score of 8 points was identified as the within-patient threshold to use to confirm a meaningful change in sleep disturbance.

Completion rates were consistent, varying between 84.6% and 99.7%. Similarly high completion rates were noted in other 12-week studies involving individuals experiencing menopause [36, 37].

The CFA provided support for the PROMIS structure, with acceptable model fit and strong relationships between the items and the general domain.

Excellent internal consistency was demonstrated using Cronbach’s alpha and McDonald’s omega. Correlations between items at baseline were sufficient, with only one low correlation between items 3 and 4 and two high correlations between items 2 and 3 and items 5 and 6. These findings supported combining the PROMIS SD-SF-8b components into a multi-item scale. In general, the item-total correlation analysis demonstrated that the PROMIS SD-SF-8b items were not redundant with sufficient relationships between the items and total scores following item omission. However, some potential redundancy was noted for some correlations, particularly those observed at week 12. Acceptable test-retest reliability was observed between baseline and week 4, with an ICC value of 0.662.

Convergent validity was generally supported by moderate correlations, although some weak correlations were observed. The highest correlations were observed with PGI-S SD and PROMIS SRI-SF-8a. This was expected as these measures are respectively used to measure sleep disturbance and sleep-related impairment. Low correlations were observed with WPAI activity impairment, presenteeism, overall work productivity loss, and absenteeism scores. These results suggest that lower associations exist between sleep disturbance and work activity and productivity. Absenteeism had the lowest correlation, possibly because individuals may attend work despite their VMS impacting their productivity. Low correlations were observed between PROMIS SD-SF-8b and the frequency or severity of VMS. This may be because the frequency and severity of VMS are analyzed using daily scores, which are not restricted to night-time VMS episodes. A low absolute correlation was found with EQ VAS, potentially because EQ VAS is a general health measure, while PROMIS SD-SF-8b focuses on sleep disturbance. Known-groups validity was supported with significant differences between the PROMIS SD-SF-8b scores and different PGI-S SD categories. As expected, PROMIS SD-SF-8b scores were higher for the more severe PGI-S SD groups, and these differences were statistically significant.

Our validity results support a previous psychometric investigation that identified the parameters to include in the PROMIS SD-SF-8b using post hoc computerized adaptive testing simulations, item discrimination parameters, and clinical judgment [17]. The convergent, discriminant, and known-groups validity findings from this previous evaluation legitimized the PROMIS SD item banks and the PROMIS SD-SF-8b measure itself.

The sensitivity to change analyses used two timepoints to provide support for the responsiveness of the PROMIS SD-SF-8b. This was demonstrated by the moderate-to-strong correlations typically observed between the change in PROMIS SD-SF-8b score and many of the PRO variables investigated (PROMIS SRI-SF-8a, PGI-S SD, PGI-C SD, MENQOL vasomotor domain, and PGI-C VMS). These findings provide evidence of sensitivity to change for the PROMIS SD-SF-8b scores when there is a change in the above measures. The weaker correlation between PROMIS SD-SF-8b score and the frequency of VMS was an unexpected finding and may be due to the reason stated above. The sensitivity results also demonstrated that significant group differences in the PROMIS SD-SF-8b scores were found using data from PGI-S SD or PGI-C SD responders or non-responders. All of these findings provide further evidence of sensitivity to change for PROMIS SD-SF-8b.

A change of ≥ 8 points in PROMIS SD-SF-8b score is recommended as the within-patient threshold required to confirm a meaningful change in sleep disturbance. Individuals who achieve a score reduction of ≥ 8 points can therefore be considered as achieving a clinically relevant improvement in sleep disturbance.

Improvements in PROMIS SD-SF-8b scores were noted at weeks 4 and 12, indicating that individuals experienced less sleep disturbance following the use of fezolinetant or placebo in SKYLIGHT 1 and 2. This finding is supported by the specific results from SKYLIGHT 2, which showed that fezolinetant 45 mg, but not fezolinetant 30 mg, significantly reduced PROMIS-assessed sleep disturbance versus placebo [15].

Overall, this study highlights the utility of the PROMIS SD-SF-8b measure to investigate sleep disturbance in individuals with moderate-to-severe VMS. In agreement with these findings, a previous qualitative study discovered that PROMIS SD-SF-8b effectively assessed constructs important to understanding sleep disturbance in this population [18].

The study does have limitations. The Cronbach’s alpha value exceeded 0.90, which can suggest some redundancy [38], although favorable CFA results were achieved. Although the possible ranges in the responder definition estimates provided by the anchors had moderate correlations, estimates may vary in different situations according to sampling variation and assessment time. These results therefore need to be confirmed in other populations with differing health conditions. The CFA was performed using baseline data with a high degree of sleep disturbance. Although unlikely, diverse results could have been generated if the data were acquired from the other timepoints investigated. In addition, the timepoints used in this analysis (baseline and weeks 4 and 12) were used to comply with the primary endpoints used in the SKYLIGHT 1 and 2 studies and were not chosen to provide meaningful psychometric results. Sleep disturbance at baseline was not an inclusion criterion for SKYLIGHT 1 and 2. Therefore, slightly different results may have been obtained if the investigation was conducted in a population with confirmed sleep disturbance. However, the mean PROMIS SD-SF-8b score at baseline was 26.80, which indicates a high degree of sleep disturbance.

## Conclusions

This study confirms the psychometric properties of the PROMIS SD-SF-8b instrument. Additionally, within-person clinically meaningful change thresholds have been established using appropriate anchors. We believe that these findings support the use of PROMIS SD-SF-8b as a fit-for-purpose instrument to measure sleep disturbance in women with moderate-to-severe VMS due to menopause.

### Supplementary Information

**Additional file 1: Further Methods.** The other patient-reported outcome (PRO) instruments included in this analysis. **Table S1.** Categories for change in anchor measure scores at weeks 4 and 12 – mean change. **Table S2.** Categories for change in anchor measure scores at weeks 4 and 12 – ROC curve. **Table S3.** Distribution of responses for PROMIS SD-SF-8b items. **Table S4.** Item-to-item correlation analysis. **Table S5.** Anchor evaluation: correlations between PROMIS SD-SF-8b total score and anchor change. **Table S6.** ROC curve analysis of PROMIS SD-SF-8b total score: responder versus non-responder.

## Data Availability

Researchers may request access to anonymized participant level data, trial level data, and protocols from Astellas sponsored clinical trials at www.clinicalstudydatarequest.com. For the Astellas criteria on data sharing see: https://clinicalstudydatarequest.com/Study-Sponsors/Study-Sponsors-Astellas.aspx.

## Abbreviations

ANCOVA: Analysis of covariance
CFA: Confirmatory factor analysis
CI: Confidence interval
FAS: Full analysis set
HT: Hormone therapy
ICC: Intraclass correlation coefficient
MENQOL: Menopause-Specific Quality of Life
NNFI: Non-normed fit index
PGI-C SD: Patient Global Impression of Change Sleep Disturbance
PGI-C VMS: Patient Global Impression of Change Vasomotor Symptoms
PGI-S SD: Patient Global Impression of Severity Sleep Disturbance
PRO: Patient-reported outcome
PROMIS: Patient-Reported Outcomes Measurement Information System
PROMIS SD-SF-8b: Patient-Reported Outcomes Measurement Information System Sleep Disturbance – Short Form 8b
PROMIS SRI-SF-8a: Patient-Reported Outcomes Measurement Information System Sleep-Related Impairment – Short Form 8a
ROC: Receiver operating characteristic
SD: Standard deviation
SEM: Standard error of measurement
SRMR: Standardized root mean residual
VAS: Visual Analog Scale
VMS: Vasomotor symptoms
WPAI-VMS: Work Productivity and Activity Impairment questionnaire specific to Vasomotor Symptoms
