# Supplementary material for: Psychometric evaluation of the PROMIS SD-SF-8b instrument in individuals experiencing vasomotor symptoms due to menopause
Source: Health Qual Life Outcomes. 2023 Nov 21;21:126. doi: 10.1186/s12955-023-02206-x (PMC10662922; doi:10.1186/s12955-023-02206-x)
Supplement: Supplementary file 1 — Additional file 1: Further Methods. The other patient-reported outcome (PRO) instruments included in this analysis. Table S1. Categories for change in anchor measure scores at weeks 4 and 12 – mean change. Table S2. Categories for change in anchor measure scores at weeks 4 and 12 – ROC curve. Table S3. Distribution of responses for PROMIS SD-SF-8b items. Table S4. Item-to-item correlation analysis. Table S5. Anchor evaluation: correlations between PROMIS SD-SF-8b total score and anchor change. Table S6. ROC curve analysis of PROMIS SD-SF-8b total score: responder versus non-responder. [file 12955_2023_2206_MOESM1_ESM.docx]

### Additional File 1

Further Methods The other patient-reported outcome (PRO) instruments included in this analysis

Table S1 Categories for change in anchor measure scores at weeks 4 and 12 – mean change

Table S2 Categories for change in anchor measure scores at weeks 4 and 12 – ROC curve

Table S3 Distribution of responses for PROMIS SD-SF-8b items

Table S4 Item-to-item correlation analysis

Table S5 Anchor evaluation: correlations between PROMIS SD-SF-8b total score and anchor change

Table S6 ROC curve analysis of PROMIS SD-SF-8b total score: responder versus non-responder

### Further Methods The other patient-reported outcome (PRO) instruments included in this analysis

For the vasomotor symptoms (VMS) episodes assessment, the participants recorded the number of VMS and the severity of each episode via an electronic PRO daily diary. The severity of an individual episode of VMS was defined as either mild (1; sensation of heat without sweating), moderate (2; sensation of heat with sweating, able to continue activity), or severe (3; sensation of heat with sweating, causing cessation of activity) [[1](#_ENREF_1), [2](#_ENREF_2)]. VMS episodes were used to assess convergent validity and sensitivity to change.

The Patient-Reported Outcomes Measurement Information System Sleep-Related Impairment – Short Form 8a (PROMIS SRI-SF-8a) is composed of eight items selected from the PROMIS sleep-related impairment item bank [[3](#_ENREF_3)]. The form encompasses self-reported perceptions of alertness, sleepiness, and tiredness during usual waking hours, as well as perceived functional impairments during wakefulness that are associated with sleep problems or impaired alertness. Higher scores are associated with more impaired sleep and range between 8 and 40. The PROMIS SRI-SF-8a was used to assess convergent validity and sensitivity to change.

The Menopause-Specific Quality of Life (MENQOL) questionnaire is a 29-item self-reported measure that assesses quality of life specific to menopause [[4](#_ENREF_4)]. The participants were asked to indicate if they have experienced any of the 29 symptoms within the past week, and then asked to rate the level of “bother” for each symptom experienced from not at all bothered (score: 0) to extremely bothered (score: 6). MENQOL contains four domains (vasomotor, psychosocial, physical, and sexual). To calculate the domain subscale scores, individual item scores were converted to a score ranging from 1 to 8 (1: not experienced, 2: bother equals 0, 8: bother equals 6). The averages of the items within the domain were calculated, with higher scores associated with increased symptom bother. MENQOL was used to assess convergent validity and sensitivity to change.

The Patient Global Impression of Severity Sleep Disturbance (PGI-S SD) instrument is a single item that measures overall severity of sleep disturbance. The participants were asked to rate the overall severity of their current sleep disturbance on a scale that ranged from no problems (score: 0) to severe problems (score: 3). The PGI-S SD was used to assess convergent validity, known-groups validity, sensitivity to change, and to derive the responder definition.

The Patient Global Impression of Change Sleep Disturbance (PGI-C SD) instrument is a single item that measures change in sleep disturbance. The participants were asked to rate the overall change from baseline to weeks 4 and 12 in their sleep disturbance on a 7-point scale ranging from much worse (score: – 3) to much better (score: + 3). The PGI-C SD was used to assess sensitivity to change and to derive the responder definition.

The Patient Global Impression of Change Vasomotor Symptoms (PGI-C VMS) instrument is a single item that assesses the overall change in VMS. The participants were asked to rate the overall change from baseline to weeks 4 and 12 in their hot flashes/night sweats on a 7-point scale ranging from much worse (score: – 3) to much better (score: + 3). The PGI-C VMS was used to assess sensitivity to change.

The EQ-5D-5 L contains five questions that measure mobility, self-care, usual activities, pain or discomfort, and anxiety or depression [[5](#_ENREF_5)]. The participants provided one response to each question from five possible response levels: no problems, slight problems, moderate problems, severe problems, and unable to/extreme problems. The EQ Visual Analog Scale (VAS) records the self-rated health of the participants on a vertical VAS from the best health you can imagine (score: 100) to the worst health you can imagine (score: 0). The EQ VAS was used to assess convergent validity and sensitivity to change.

The Work Productivity and Activity Impairment questionnaire specific to Vasomotor Symptoms (WPAI-VMS) is a six-item PRO measure that examines VMS-related work productivity and activity in the preceding 7 days [[6](#_ENREF_6)]. The questionnaire contains four domains, absenteeism (the percentage of work time missed because of VMS in the past 7 days), presenteeism (the percentage of impairment experienced while at work because of VMS in the past 7 days), overall work productivity loss (overall work impairment measured by combining absenteeism and presenteeism to determine the total percentage of missed time), and activity impairment (the percentage of impairment in daily activities because of VMS in the past 7 days). WPAI-VMS outcomes are expressed as impairment percentages, with higher numbers indicating greater impairment and less productivity. The WPAI-VMS was used to assess convergent validity.

### Table S1 Categories for change in anchor measure scores at weeks 4 and 12 – mean change

| **Anchor** | **Anchor category** | **Definition** |
| --- | --- | --- |
| PGI-C SD at weeks 4/12 – all categories | Much better | Score of + 3 (“much better”) |
|  | Moderately better | Score of + 2 (“moderately better”) |
|  | A little better | Score of + 1 (“a little better”) |
|  | No change | Score of 0 (“no change”) |
|  | A little worse | Score of – 1 (“a little worse”) |
|  | Moderately worse | Score of – 2 (“moderately worse”) |
|  | Much worse | Score of – 3 (“much worse”) |
| PGI-C SD at weeks 4/12 – collapsed categories 1 | Much better or moderately better | Score of + 3 or + 2 (“much better” or “moderately better”) |
|  | Moderately worse or much worse | Score of – 2 or – 3 (“moderately worse” or “much worse”) |
| PGI-C SD at weeks 4/12 – collapsed categories 2 | Improvement | ≥ 1-point increase (little, moderately, or much better levels) |
|  | No change | 0 points (no change) |
|  | Worsening | ≥ 1-point decrease (little, moderately, or much worse levels) |
| PGI-S SD change at weeks 4/12 – all categories | Improved 3 points | 3-point decrease (a lot of improvement) |
|  | Improved 2 points | 2-point decrease (moderate improvement) |
|  | Improved 1 point | 1-point decrease (little improvement) |
|  | No change | 0 points (no change) |
|  | Worsened 1 point | 1-point increase (little worsening) |
|  | Worsened 2 points | 2-point increase (moderate worsening) |
|  | Worsened 3 points | 3-point increase (a lot of worsening) |
| PGI-S SD change at weeks 4/12 – collapsed categories | Improved ≥ 2 points | ≥ 2-point decrease (moderate, a lot of improvement) |
|  | Worsened ≥ 2 points | ≥ 2-point increase (moderate, a lot of worsening) |

Change from baseline was analyzed at weeks 4 and 12 for the PGI-S SD anchor and response was analyzed at weeks 4 and 12 for the PGI-C SD anchor

*PGI-C SD* Patient Global Impression of Change Sleep Disturbance, *PGI-S SD* Patient Global Impression of Severity Sleep Disturbance

### Table S2 Categories for change in anchor measure scores at weeks 4 and 12 – ROC curve

| **Anchor** | **Anchor category** | **Definition** |
| --- | --- | --- |
| PGI-C SD at weeks 4/12 – collapsed categories (ROC) | Responder | “Moderately better” or “much better” levels (i.e., score of + 2 and + 3) |
|  | Non-responders | All other levels (i.e., score of + 1, 0, – 1, – 2, and – 3) |
| PGI-S SD change at weeks 4/12 – collapsed categories (ROC) | Responder | ≥ 2-point improvement/decrease (moderate, a lot of improvement) |
|  | Non-responders | All other changes (i.e., 1-point decrease [little improvement] and ≥ 0-point increase [no change, little, moderate, and a lot of worsening]) |

Change from baseline was analyzed at weeks 4 and 12 for the PGI-S SD anchor and response was analyzed at weeks 4 and 12 for the PGI-C SD anchor

*PGI-C SD* Patient Global Impression of Change Sleep Disturbance, *PGI-S SD* Patient Global Impression of Severity Sleep Disturbance, *ROC* Receiver operating characteristic

### Table S3 Distribution of responses for PROMIS SD-SF-8b items

| **Item/timepoint** | **FAS (*N* = 1022) Item response options, *n* (%)** | | | | | |
| --- | --- | --- | --- | --- | --- | --- |
|  | ***N*** | **1** | **2** | **3** | **4** | **5** |
| Item 1: my sleep was restless | |  |  |  |  |  |
| Baseline | 1019 | 60 (5.9) | 156 (15.3) | 272 (26.7) | 339 (33.3) | 192 (18.8) |
| Week 4 | 933 | 113 (12.1) | 287 (30.8) | 288 (30.9) | 181 (19.4) | 64 (6.9) |
| Week 12 | 865 | 133 (15.4) | 267 (30.9) | 265 (30.6) | 136 (15.7) | 64 (7.4) |
| Item 2: I was satisfied with my sleep | |  |  |  |  |  |
| Baseline | 1019 | 38 (3.7) | 127 (12.5) | 333 (32.7) | 263 (25.8) | 258 (25.3) |
| Week 4 | 933 | 79 (8.5) | 198 (21.2) | 306 (32.8) | 210 (22.5) | 140 (15.0) |
| Week 12 | 865 | 90 (10.4) | 170 (19.7) | 305 (35.3) | 192 (22.2) | 108 (12.5) |
| Item 3: my sleep was refreshing | |  |  |  |  |  |
| Baseline | 1019 | 37 (3.6) | 130 (12.8) | 295 (28.9) | 285 (28.0) | 272 (26.7) |
| Week 4 | 933 | 65 (7.0) | 196 (21.0) | 289 (31.0) | 221 (23.7) | 162 (17.4) |
| Week 12 | 865 | 78 (9.0) | 171 (19.8) | 297 (34.3) | 186 (21.5) | 133 (15.4) |
| Item 4: had difficulty falling asleep | |  |  |  |  |  |
| Baseline | 1019 | 225 (22.1) | 241 (23.7) | 244 (23.9) | 199 (19.5) | 110 (10.8) |
| Week 4 | 933 | 280 (30.0) | 274 (29.4) | 211 (22.6) | 121 (13.0) | 47 (5.0) |
| Week 12 | 865 | 276 (31.9) | 258 (29.8) | 188 (21.7) | 99 (11.4) | 44 (5.1) |
| Item 5: had trouble staying asleep | |  |  |  |  |  |
| Baseline | 1019 | 35 (3.4) | 107 (10.5) | 323 (31.7) | 377 (37.0) | 177 (17.4) |
| Week 4 | 933 | 61 (6.5) | 208 (22.3) | 364 (39.0) | 227 (24.3) | 73 (7.8) |
| Week 12 | 865 | 56 (6.5) | 214 (24.7) | 341 (39.4) | 187 (21.6) | 67 (7.7) |
| Item 6: had trouble sleeping |  |  |  |  |  |  |
| Baseline | 1019 | 41 (4.0) | 145 (14.2) | 356 (34.9) | 333 (32.7) | 144 (14.1) |
| Week 4 | 933 | 87 (9.3) | 230 (24.7) | 368 (39.4) | 184 (19.7) | 64 (6.9) |
| Week 12 | 865 | 78 (9.0) | 235 (27.2) | 332 (38.4) | 171 (19.8) | 49 (5.7) |
| Item 7: got enough sleep |  |  |  |  |  |  |
| Baseline | 1019 | 45 (4.4) | 163 (16.0) | 373 (36.6) | 337 (33.1) | 101 (9.9) |
| Week 4 | 933 | 61 (6.5) | 279 (29.9) | 344 (36.9) | 191 (20.5) | 58 (6.2) |
| Week 12 | 865 | 68 (7.9) | 248 (28.7) | 345 (39.9) | 163 (18.8) | 41 (4.7) |
| Item 8: my sleep quality was… | |  |  |  |  |  |
| Baseline | 1019 | 31 (3.0) | 160 (15.7) | 450 (44.2) | 293 (28.8) | 85 (8.3) |
| Week 4 | 933 | 50 (5.4) | 267 (28.6) | 423 (45.3) | 140 (15.0) | 53 (5.7) |
| Week 12 | 865 | 58 (6.7) | 253 (29.2) | 390 (45.1) | 129 (14.9) | 35 (4.0) |

Higher scores indicate worse sleep disturbance. Percentages are based on the number of individuals in the FAS with non-missing values

*FAS* Full analysis set, *PROMIS SD-SF-8b* Patient-Reported Outcomes Measurement Information System Sleep Disturbance – Short Form 8b

### Table S4 Item-to-item correlation analysis

| **Item** | **Item 1** | **Item 2** | **Item 3** | **Item 4** | **Item 5** | **Item 6** | **Item 7** | **Item 8** |
| --- | --- | --- | --- | --- | --- | --- | --- | --- |
| Item 1: my sleep was restless | - | 0.676 | 0.604 | 0.463 | 0.693 | 0.703 | 0.577 | 0.725 |
| Item 2: I was satisfied with my sleep |  | - | 0.829 | 0.414 | 0.636 | 0.658 | 0.677 | 0.778 |
| Item 3: my sleep was refreshing |  |  | - | 0.357 | 0.562 | 0.600 | 0.650 | 0.743 |
| Item 4: had difficulty falling asleep |  |  |  | - | 0.441 | 0.582 | 0.464 | 0.503 |
| Item 5: had trouble staying asleep |  |  |  |  | - | 0.806 | 0.635 | 0.717 |
| Item 6: had trouble sleeping |  |  |  |  |  | - | 0.636 | 0.762 |
| Item 7: got enough sleep |  |  |  |  |  |  | - | 0.755 |
| Item 8: my sleep quality was… |  |  |  |  |  |  |  | - |

Polychoric correlation coefficients are shown

### Table S5 Anchor evaluation: correlations between PROMIS SD-SF-8b total score and anchor change

| **Visit** | **PGI-S SD change** | | **PGI-C SD change categories** | |
| --- | --- | --- | --- | --- |
|  | ***N*** | ***r*** | ***N*** | ***r*** |
| Baseline to week 4 | 932 | 0.606 | 931 | 0.572 |
| Baseline to week 12 | 864 | 0.616 | 862 | 0.526 |

Spearman correlations are shown

*PGI-C SD* Patient Global Impression of Change Sleep Disturbance, *PGI-S SD* Patient Global Impression of Severity Sleep Disturbance, *PROMIS SD-SF-8b* Patient-Reported Outcomes Measurement Information System Sleep Disturbance – Short Form 8b

### Table S6 ROC curve analysis of PROMIS SD-SF-8b total score: responder versus non-responder

| **Timepoint** | **Anchor** | **AUC** | **Sensitivity** | **Specificity** | **Criterion** |
| --- | --- | --- | --- | --- | --- |
| Baseline to week 4 | PGI-S SD | 0.84 | 0.73 | 0.78 | – 8.00 |
|  | PGI-C SD | 0.79 | 0.79 | 0.66 | – 4.00 |
| Baseline to week 12 | PGI-S SD | 0.83 | 0.80 | 0.72 | – 7.00 |
|  | PGI-C SD | 0.76 | 0.74 | 0.64 | – 4.00 |

For PGI-S SD, responders were defined by a ≥ 2-point decrease (moderate improvement or a lot of improvement), with all other levels defined as non-responders. For PGI-C SD, responders were defined by “moderately better” or “much better” levels, with all other levels defined as non-responders

*AUC* Area under the curve, *PGI-C SD* Patient Global Impression of Change Sleep Disturbance, *PGI-S SD* Patient Global Impression of Severity Sleep Disturbance, *PROMIS SD-SF-8b* Patient-Reported Outcomes Measurement Information System Sleep Disturbance – Short Form 8b, *ROC* Receiver operating characteristic

### References

1. U.S. Department of Health and Human Services, Food and Drug Administration, Center for Drug Evaluation and Research (CDER). Guidance for industry. Estrogen and estrogen/progestin drug products to treat vasomotor symptoms and vulvar and vaginal atrophy symptoms — recommendations for clinical evaluation. Draft guidance. January 2003. <https://www.fda.gov/media/71359/download>. Accessed 20 Sept 2023.

2. Committee for Medicinal Products for Human Use (CHMP). Guideline on clinical investigation of medicinal products for hormone replacement therapy of oestrogen deficiency symptoms in postmenopausal women. 13 October 2005. <https://www.ema.europa.eu/en/documents/scientific-guideline/guideline-clinical-investigation-medicinal-products-hormone-replacement-therapy-oestrogen-deficiency_en.pdf>. Accessed 20 Sept 2023.

3. Patient-Reported Outcomes Measurement Information System. Sleep-related impairment. <https://www.healthmeasures.net/images/PROMIS/manuals/PROMIS_Sleep-Related_Impairment_Scoring_Manual.pdf>. Accessed 20 Sept 2023.

4. Hilditch JR, Lewis J, Peter A, van Maris B, Ross A, Franssen E, et al. A menopause-specific quality of life questionnaire: development and psychometric properties. Maturitas. 1996;24(3):161-75.

5. Herdman M, Gudex C, Lloyd A, Janssen M, Kind P, Parkin D, et al. Development and preliminary testing of the new five-level version of EQ-5D (EQ-5D-5L). Qual Life Res. 2011;20(10):1727-36.

6. Reilly MC, Zbrozek AS, Dukes EM. The validity and reproducibility of a work productivity and activity impairment instrument. Pharmacoeconomics. 1993;4(5):353-65.
